# Clinical implications of DNA methylation-based integrated classification of histologically defined grade 2 meningiomas

**DOI:** 10.1186/s40478-024-01739-6

**Published:** 2024-05-08

**Authors:** Felix Ehret, Eilís Perez, Daniel Teichmann, Sandra Meier, Carola Geiler, Cosmas Zeus, Helene Franke, Siyer Roohani, David Wasilewski, Julia Onken, Peter Vajkoczy, Leonille Schweizer, David Kaul, David Capper

**Affiliations:** 1 Charité – Universitätsmedizin Berlin, Corporate Member of Freie Universität Berlin and Humboldt-Universität zu Berlin, Department of Radiation Oncology, Berlin, Germany; 2 Charité – Universitätsmedizin Berlin, Berlin, Germany; German Cancer Consortium (DKTK), partner site Berlin, and German Cancer Research Center (DKFZ), Heidelberg, Germany; 3 Charité – Universitätsmedizin Berlin, Corporate Member of Freie Universität Berlin and Humboldt-Universität zu Berlin, Department of Neuropathology, Berlin, Germany; 4 Charité – Universitätsmedizin Berlin, Corporate Member of Freie Universität Berlin and Humboldt-Universität zu Berlin, Berlin School of Integrative Oncology (BSIO), Berlin, Germany; 5 Berlin Institute of Health at Charité – Universitätsmedizin Berlin, BIH Biomedical Innovation Academy, BIH Charité Junior Clinician Scientist Program, Berlin, Germany; 6 Charité – Universitätsmedizin Berlin, Corporate Member of Freie Universität Berlin and Humboldt-Universität zu Berlin, Department of Neurosurgery, Berlin, Germany; 7 Institute of Neurology (Edinger Institute), University Hospital Frankfurt, Goethe University Frankfurt, Frankfurt Am Main, Germany

**Keywords:** Atypical meningioma, Meningioma, DNA methylation, Risk score, Radiotherapy

## Abstract

The combination of DNA methylation analysis with histopathological and genetic features allows for a more accurate risk stratification and classification of meningiomas. Nevertheless, the implications of this classification for patients with grade 2 meningiomas, a particularly heterogeneous tumor entity, are only partially understood. We correlate the outcomes of histopathologically confirmed grade 2 meningioma with an integrated molecular-morphologic risk stratification and determine its clinical implications. Grade 2 meningioma patients treated at our institution were re-classified using an integrated risk stratification involving DNA methylation array-based data, copy number assessment and TERT promoter mutation analyses. Grade 2 meningioma cases according to the WHO 2021 criteria treated between 2007 and 2021 (n = 100) were retrospectively analyzed. The median clinical and radiographic follow-up periods were 59.8 and 54.4 months. A total of 38 recurrences and 17 deaths were observed. The local control rates of the entire cohort after 2-, 4-, and 6-years were 84.3%, 68.5%, and 50.8%, with a median local control time of 77.2 months. The distribution of the integrated risk groups were as follows: 31 low, 54 intermediate, and 15 high risk cases. In the multivariable Cox regression analysis, integrated risk groups were significantly associated with the risk of local recurrence (hazard ratio (HR) intermediate: 9.91, HR high-risk: 7.29, *p* < 0.01). Gross total resections decreased the risk of local tumor progression (HR gross total resection: 0.19, *p* < 0.01). The comparison of 1p status and integrated risk groups (low vs. intermediate/high) revealed nearly identical local control rates within their respective subgroups. In summary, only around 50% of WHO 2021 grade 2 meningiomas have an intermediate risk profile. Integrated molecular risk stratification is crucial to guide the management of patients with grade 2 tumors and should be routinely applied to avoid over- and undertreatment, especially concerning the use of adjuvant radiotherapy.

**Supplementary Information:**

The online version contains supplementary material available at 10.1186/s40478-024-01739-6.

## Introduction

Meningiomas are the most common brain tumors in adults [1]. While most meningiomas have a benign nature, approximately 20% display an aggressive clinical behavior with a distinct tendency to recur [1, 2]. Treatment options in aggressive meningiomas comprise surgery, adjuvant radiotherapy, and experimental therapies within the setting of clinical trials [2, 3]. World Health Organization (WHO) grade 2 tumors represent an especially heterogeneous subgroup of meningiomas, with a considerable variety concerning their clinical course after surgery and radiotherapy [4–7]. The ideal management of this tumor in regard to the use of adjuvant radiotherapy after gross total resection, target delineation, safety margins, and prescription dose remains an active area of investigation with ongoing trials such as the EORTC-1308/ROAM and NRG-BN003 aiming to provide further evidence [8]. Recent advances in molecular profiling, with a particular focus on DNA methylation analyses, have enabled an improved risk stratification of meningiomas in general [9–13]. The development of an integrated molecular-morphological classification has the potential to refine and individualize the treatment [10]. However, the clinical implications for the management of grade 2 meningiomas, which poses a common challenge in neuro-oncology due to their heterogeneous behavior, are less defined, given the lack of dedicated analyses. Herein, we perform a comprehensive analysis of grade 2 meningiomas, assessing their global DNA methylation profiles and telomerase reverse transcriptase (TERT) promoter mutation status, determining their molecular risk group, and correlating this to the clinical course with subsequent analysis of treatment decision-making.

## Materials and methods

### Patient cohort and follow-up

In this single-institutional retrospective study, we screened histologically defined WHO grade 2 meningiomas treated at our institution between 2007 and 2021. Inclusion criteria comprised patients with available archived formalin-fixed paraffin-embedded (FFPE) tumor tissue and clinical as well as radiographic follow-up data of at least one year after surgical resection. All tumors were reassessed by a board certified neuropathologist to confirm a tumor grade 2 according to the 2021 WHO Classification of Tumors of the Central Nervous System [14]. Cases with primary resections and treatment for tumor recurrences were allowed. Patient, tumor, and treatment characteristics were extracted from medical records and patient files. Variables of interest included patient demographics (age, sex), tumor characteristics (grading, histopathological subtype, location, size), treatment modalities (treatment indication, Simpson grade, radiotherapy), local tumor control, and date of death [15]. Gross total resection was defined as Simpson grades I, II, and III, while subtotal resection included all cases with Simpson grade IV and V [15]. The resection status was determined by the surgical notes and, when available, postoperative imaging. The integrated molecular-morphological risk score was calculated as previously described and incorporates WHO grading, copy number variation (CNV), including 1p, 6q, and 14q losses, and the methylation family [10]. Clinical follow-up was calculated from the day of surgery to the last clinical contact. Radiographic follow-up was defined as the period from surgery to the last available imaging with contrast-enhanced magnetic resonance imaging (MRI). Local control was defined as the absence of tumor recurrence or progression such as tumor volume increase, at the initial tumor site. MRI was assessed by a board certified neuroradiologist to evaluate local control and classify local failures. The local control rate was calculated as the proportion of patients who exhibited no evidence of local tumor recurrence or progression during the follow-up period utilizing the Kaplan–Meier estimator. The progression-free survival (PFS) was calculated from the time of surgery to local tumor progression or death of any cause. Overall survival (OS) was defined as the period between surgical resection and death of any cause. Censoring for PFS and OS occurred on the last day of available clinical follow-up, for local control on the last day of available radiographic follow-up.

### Molecular and statistical analyses

DNA methylation analysis was performed using the 850 k EPIC Illumina Infinium Methylation Array (Illumina, USA). DNA was extracted from the FFPE tumor samples using the Maxwell RSC FFPE Plus DNA Purification Kit (kit number AS1720) (Promega, USA). After bisulfite conversion using the EpiTect Fast Bisulfite Conversion Kit (Qiagen, Netherlands), the Infinium HD FFPE DNA Restore Kit was used for DNA restoration. The beadarrays were scanned on the iScan system (Illumina, USA). The unprocessed output data (idat files) from the iScan reader were checked for general quality measures according to the manufacturer. Raw data obtained from the analysis platform were processed using the R package minfi, version 1.4.0 [16]. The rgSets of 850 k EPIC array were merged with a 450 k array dataset comprising 148 previously analyzed meningioma samples as a reference cohort [9]. A combined rgSet in 450 k output format was generated using the set of overlap probes on both array types. The combined dataset was then subjected to quality control measures, including filtering and functional normalization procedures, to ensure data integrity and comparability across samples as previously described [17]. After filtering and normalization, the variance in the data set was estimated by applying standard deviation over the β-values of all remaining probes. The top 25,000 most variable probes were kept for dimensionality reduction using the Rtsne package, version 0.15 [18]. A Pearson distance matrix was used as the input object, theta of 0.5, perplexity of 30, and all other default parameters were used. Visualizations were generated with the ggplot2 package version 3.3.5 [19]. DNA methylation-based classification was done via MolecularNeuropathology.org, using the brain tumor classifier v12.5. The meningioma classifier v2.4 was used for exploration of matching agreement. The highest score was used for classification, no cut-offs were applied. All tumors underwent TERT promoter mutation analysis with Sanger sequencing, followed by an assessment of hotspot mutations (C228/C250). Assessment of homozygous deletion of cyclin-dependent kinase inhibitors 2A/B (CDKN2A/B) was based on CNV profiles generated using the conumee R package [20]. Chromosomal arm deletions were assessed using the CNV profile as previously reported [21]. Visualization of methylation families was done utilizing t-distributed stochastic neighbor embedding (t-SNE). Descriptive statistics, including frequencies, ranges, and medians, were used to summarize patient and tumor characteristics. Additionally, multivariable Cox proportional hazards models were utilized to evaluate the association of relevant clinical variables on the local tumor control, PFS, and OS. Variable selection was done a priori and based on the most relevant risk factors known [10, 15]. The proportional hazards assumptions were tested using global tests based on Schoenfeld residuals and visual assessment of log–log plots. The goodness of fit of the created models was assessed with the concordance index (Harrell’s C). Statistical significance was defined as *p*-values ≤ 0.05. Statistical analysis was performed using STATA MP 17.0 (StataCorp, USA). Figures were created with STATA MP 17.0 and R (R Foundation for Statistical Computing, Austria). The study was approved by the local institutional review board (EA2/059/21).

## Results

### Patient cohort

**Fig. 1:**
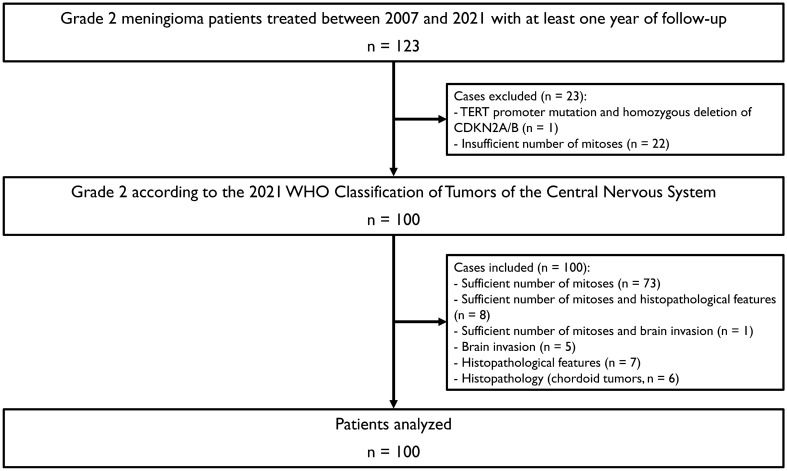
Patient cohort flowchart with details concerning included and excluded cases

A total of 123 patients treated between 2007 and 2021 were screened (Fig. 1). Sixty-nine patients were diagnosed between 2007 (4th WHO CNS tumor classification) and 2016, the remaining 54 between 2016 and 2021 (4th edition update WHO CNS tumors classification) [22, 23]. After reassessment of tumor grading according to the WHO 2021 classification, 23 cases did not fulfill current criteria. The final study set thus comprised 100 cases (Fig. 1). The exclusion of 23 cases was primarily based due to changes of the cutoff of mitotic rate in WHO 2021 (22/23, 95.6%). One tumor was found to have a TERT promoter mutation as well as a homozygous deletion of CDKN2A/B. The median radiographic and clinical follow-up for the final cohort were 54.4 (range: 12.3–155.2) and 59.8 months (range: 12.4–155.2), respectively. The majority of analyzed tumors were atypical meningiomas (94/100, 94.0%) and resected as part of the primary management at first diagnosis (97/100, 97.0%). The median age at surgery was 59.1 years (range: 20.9–86.9). Most tumors were located around the convexity (66/100, 66.0%) and at the skull base (32/100, 32.0%), with only two cases (2.0%) located in the posterior horn of the lateral ventricles. Gross total and subtotal resection were achieved in 78/100 (78.0%) and 14/100 (14.0%) patients, respectively. Fifteen patients of 100 (15/100, 15.0%) received adjuvant radiotherapy. Seven of these patients (7/15, 46.7%) had a subtotal resection resulting in postoperative treatment. The remaining eight treatment decisions (8/15, 53.3%) were based on individual preferences.

### Clinical outcomes and molecular analyses

**Fig. 2:**
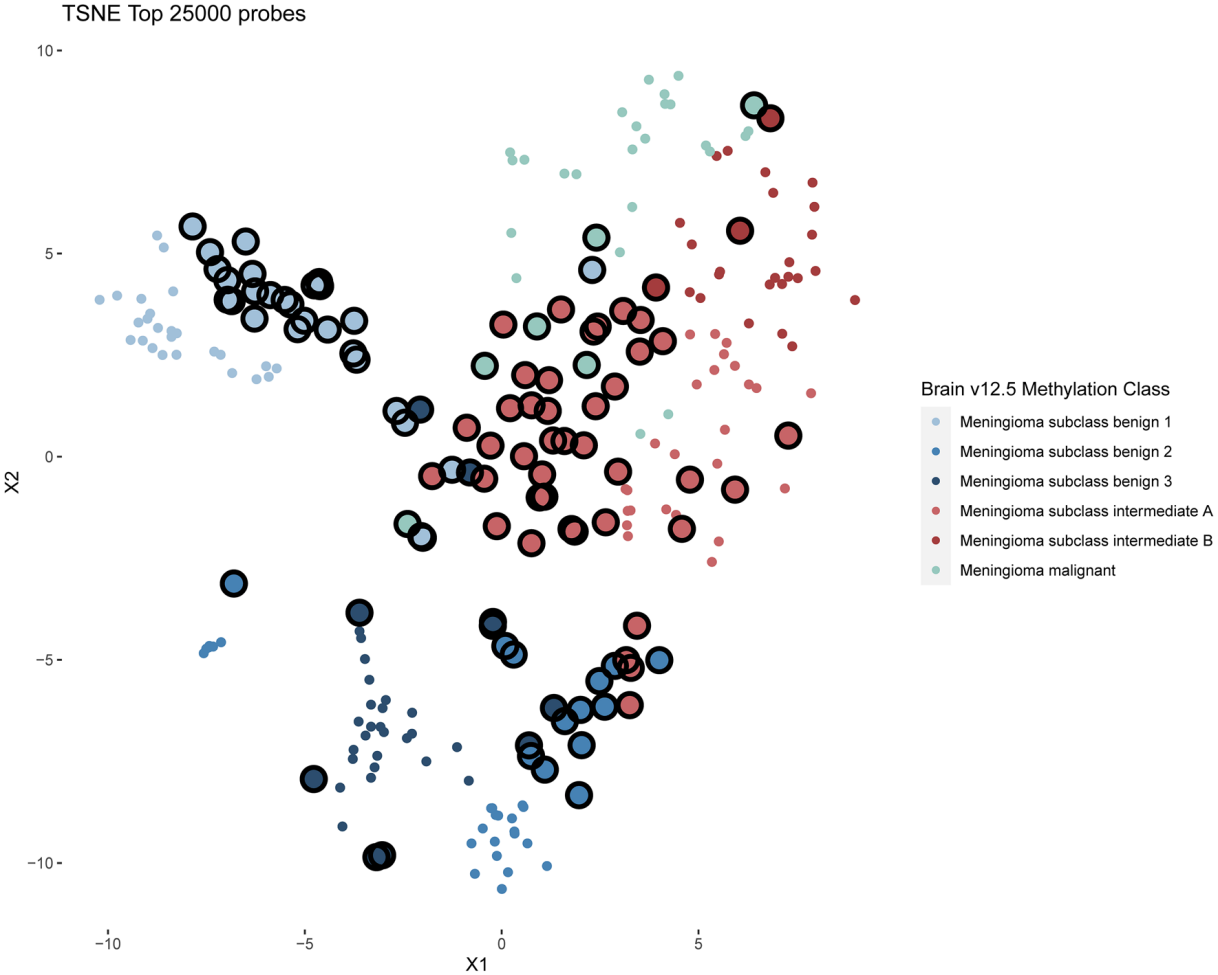
t-SNE showing the methylation families as determined by the brain tumor classifier v12.5. Enlarged dots represent the grade 2 meningiomas analyzed in this study. Small dots show a reference cohort of 148 cases previously analyzed for better visualization and grouping [9]

**Fig. 3:**
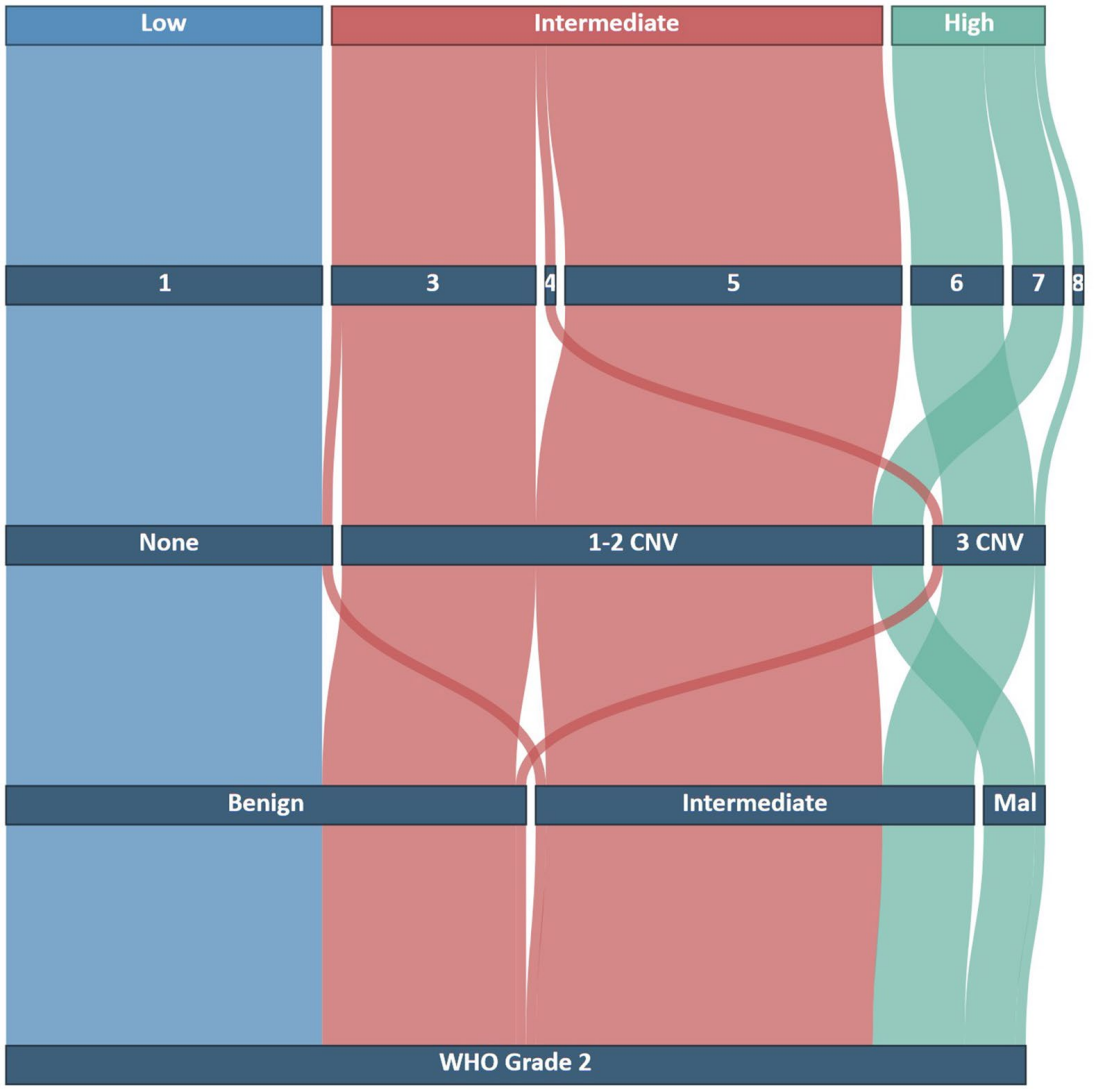
Alluvial plot for the assessment of the integrated molecular-morphological risk group using the brain tumor classifier v12.5. The starting layer (bottom) is represented by the WHO grading based on the histopathological examination. All analyzed tumors underwent methylation analyses with subsequent allocation to the three methylation families (benign, intermediate, and malignant (Mal), second layer). To calculate the risk score, CNV assessment of 1p, 6q, and 14 q was additionally required (third layer). After calculation of the risk score (fourth layer), the final risk group assessment was done (fifth layer)

**Fig. 4:**
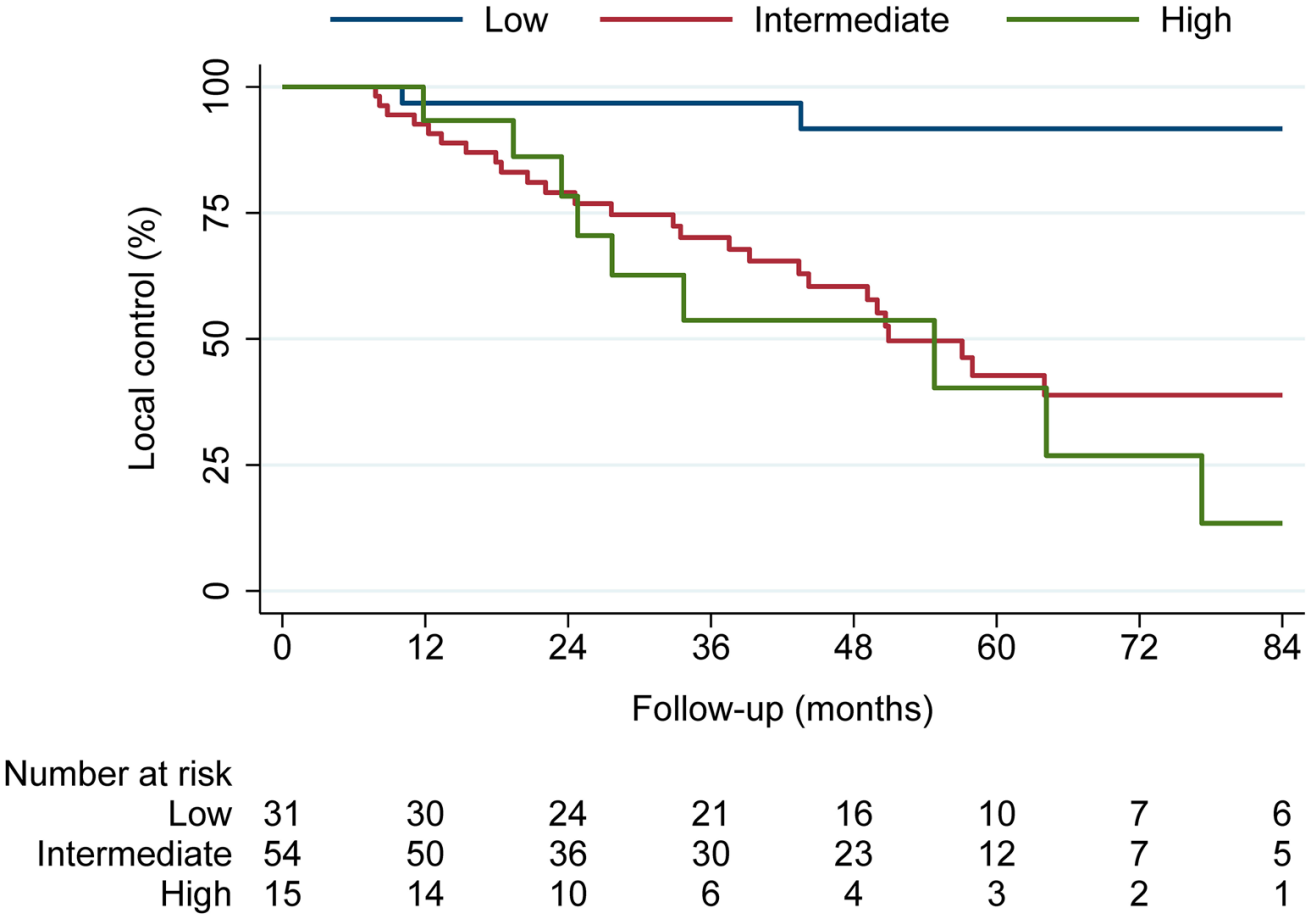
Local control rates based on the integrated molecular-morphological risk score utilizing the brain tumor classifier v12.5. Intermediate and high risk tumors show a significantly elevated risk for local failures while low risk meningiomas display a durable local control

A total of 38 recurrences (38.0%) and 17 deaths (17.0%) were observed. The local control rates of the entire cohort after 2-, 4-, and 6-years were 84.3%, 68.5%, and 50.8%, with a median local control time of 77.2 months (95% confidence interval: 50.9—not available). The most common methylation family according to the brain tumor classifier version 12.5 was benign (51/100, 51.0%), followed by intermediate (43/100, 43.0%) and malignant (6/100, 6.0%) (Fig. 2). The patient and tumor characteristics as well as follow-up information are summarized in Table 1. Assessment of CNV revealed a total of 60 1p (60.0%), 30 6q (30.0%), and 35 14q (35.0%) losses (example case in Additional file 1: Fig. S1). Calculation of the integrated molecular-morphological classification led mostly to intermediate risk tumors (54/100, 54.0%), followed by low (31/100, 31.0%) and high risk (15/100, 15.0%) meningiomas (Fig. 3). The number of 1p losses increased from low risk (0/31, 0.0%), intermediate risk (45/54, 83.3%) to high risk tumors (15/15, 100.0%). The local control rates between risk groups varied markedly and are shown in Fig. 4. The median times of local control for the intermediate and high risk groups were 50.9 and 54.7 months, respectively. The median local control time for the low risk group was not reached during the available follow-up period. Comparison of 1p status and integrated risk groups (low vs. intermediate/high) demonstrated nearly identical local control rates among both corresponding subgroups (Additional file 1: Fig. S2).

**Table 1:** Patient and tumor characteristics with follow-up information

Number of patients	100					
Sex (male/female)	47			53		
	Median			Range		
Age at surgery (years)	59.1			20.9–86.9		
Radiographic follow-up (months)	54.4			12.3–155.2		
Clinical follow-up (months)	59.8			12.4–155.2		
Methylation class score brain tumor classifier v12.5	0.94			0.37–1.0		
Histology	Atypical			Chordoid		
Number of patients	94			6		
Primary/recurrence	Primary: 97			Recurrence: 3		
Simpson grade	I	II	III	IV	V	NA
Number of patients	43	30	5	14	0	8
Tumor location	Convexity/Falx		Skull base		Ventricle	
Number of patients	66		32		2*	
Adjuvant radiotherapy	Yes			No		
Number of patients	15			85		
Local recurrences observed	38					
Deaths observed	17					

*NA* not available

*Both located in the posterior horn of the lateral ventricle

The multivariable Cox regression analyses confirmed the integrated risk group assignment as the predominant factor influencing the local control (hazard ratio intermediate risk group: 9.91, hazard ratio high risk group: 7.29, *p* = 0.002, concordance index: 0.73, Table 2). A gross total resection decreased the risk of local tumor progression (hazard ratio: 0.19, *p* = 0.001, Table 2, Additional file 1: Fig. S3). The use of adjuvant radiotherapy was not formally associated with an improved local control (hazard ratio: 0.40, *p* = 0.09, Table 2, Additional file 1: Fig. S4).

**Table 2:** Multivariable Cox regression analyses for local control and progression-free survival. Both analyses demonstrate an elevated risk for local failures and progression based on intermediate and high risk groups per integrated molecular-morphological risk score. Gross total resection was associated with a decreased risk for local recurrences and prolonged PFS

Variable	Hazard ratio	Confidence interval (95%)	*p*-value
**Local control**			
Integrated risk group			
Low	Reference		0.002
Intermediate	9.91	2.34–41.9	
High	7.29	1.48–35.7	
Resection status			
Subtotal resection	Reference		0.001
Gross total resection	0.19	0.07–0.51	
Unknown	0.31	0.08–1.19	
Adjuvant radiotherapy			
No	Reference		0.09
Yes	0.40	0.13–1.18	
**Progression-free survival**			
Integrated risk group			
Low	Reference		0.006
Intermediate	4.38	1.53–12.5	
High	4.75	1.37–16.4	
Resection status			
Subtotal resection	Reference		0.01
Gross total resection	0.36	0.16–0.79	
Unknown	0.67	0.21–2.07	
Adjuvant radiotherapy			
No	Reference		0.26
Yes	0.58	0.23–1.48	
Age (in years)	1.01	0.99–1.03	0.21

The PFS rates of the full cohort after 2-, 4-, and 6-years were 83.6%, 68.1%, and 48.5%, with a median PFS time of 65.5 months (95% confidence interval: 54.7–122.5) (Additional file 1: Fig. S5). Comparable to the local control times, the PFS rates notably varied between the integrated risk groups (Additional file 1: Fig. S6). The median PFS time for the intermediate and high risk groups were 57.1 and 54.7 months, respectively. The median PFS for the low risk group was not reached. The integrated risk groups also showed a significant association with PFS (hazard ratio intermediate risk group: 4.38, hazard ratio high risk group: 4.75, *p* = 0.006, concordance index: 0.72, Table 2). In addition, gross total resection led to a decreased risk of progression (hazard ratio: 0.36, *p* = 0.01, Table 2). The multivariable analysis for OS revealed a gross total resection to be associated with a decreased risk of death (hazard ratio: 0.11, *p* = 0.004, concordance index: 0.88, full data not shown), while older age increased the risk (hazard ratio: 1.14, *p* < 0.001) (Additional file 1: Fig. S7). The proportional hazards assumptions were fulfilled for all variables and investigated endpoints.

### Clinical implications of the integrated molecular-morphological risk score

Of the 15 patients receiving adjuvant radiotherapy, four (4/15, 26.6%) had a low risk tumor according to the integrated molecular-morphological risk classifier. None of these four cases with radiotherapy suffered from local tumor progression during the available follow-up (Additional file 1: Fig. S8). The 85 patients without radiotherapy comprised 13 high risk tumors (13/85, 15.2%) and 45 intermediate risk cases (45/85, 52.9%) (Additional file 1: Figs. S9 and 10). No clear benefit can be derived from the use of postoperative radiotherapy in intermediate tumors. In the high risk subgroup, patients without adjuvant radiotherapy had a poor local control with a median time of less than three years. Both patients with postoperative treatments, however, remained free of local tumor progression. Assuming at least a moderate local control benefit from adjuvant radiotherapy for intermediate and high risk tumors, a total of 58 out of 85 patients (68.2%) without postoperative radiotherapy would have been potential candidates for radiation therapy. Conversely, 26.6% of patients (4/15) with a low risk group tumor could have been potentially spared immediate adjuvant radiotherapy due to the low risk of local tumor progression, assuming a marginal benefit of radiation therapy in low risk cases (Additional file 1: Fig. S8). In summary, besides the resection status, the information of the integrated molecular-morphological risk classifier could have informed the treatment decision making process in 62 cases (62/100, 62.0%).

## Discussion

Herein, we report our institutional experience with the recently introduced integrated molecular-morphological risk classifier for meningiomas [10]. We specifically focused our analysis on grade 2 meningiomas as they comprise a particularly challenging entity with a board variety of clinical courses and limited in-depth data available [4, 6, 7, 24]. The current management of grade 2 meningiomas is primarily based on a safe surgical resection [2]. Then, however, controversies remain regarding the role of adjuvant radiotherapy, particularly in the setting of a gross total resection. According to the recommendations of the European Association of Neuro-Oncology, adjuvant radiotherapy is advised for patients with subtotal resection and an option besides observation after gross total resection [2]. In the light of our observed results, several topics should be addressed. Besides the logistical, financial, and technical requirements of the classifier, i.e., lab infrastructure, technicians, and consumables, its clinical relevance is of particular importance as seen in the reported results. As histological grading may ideally reflect the biological behavior and aggressiveness of tumors, one would assume that histologically defined grade 2 meningiomas mostly display a disease course comparable to tumors that match the criteria of the intermediate risk group as defined by the integrated molecular-morphological risk classifier [10].

The likelihood of patients in this cohort having a meningioma with an intermediate risk profile as suggested by a grade 2 histology, however, was only around 50%. This finding has significant implications for the management of such tumors and highlights the urgent need for the routine implementation of integrated and molecular classifiers. Histopathological grading alone is not sufficient to adequately predict the biological behavior and to guide individual treatment decision-making. The time of local control differed substantially between all three integrated risk groups, most notably between the low risk group (5-year rate: 91.8%) and high risk group (5-year rate: 40.2%). The multivariable Cox regression confirmed the importance of the risk group assignment for local control. Such differences are critical and information from the integrated molecular-morphological risk score will substantially help to counsel patients and guide treatment-related discussions. Considering that all tumors were classified as grade 2 meningiomas according to the current WHO tumor classification, the considerable variety of observed disease courses and risk of disease progression cannot be unmasked by histopathology alone.

This may be of particular relevance for treatment individualization and the implementation of postoperative radiotherapy, an ongoing topic of debate with several, partially contradicting studies published [6, 7, 24]. Previous work investigating the role of radiotherapy in grade 2 meningiomas did not routinely incorporate molecular characteristics, such as methylation profiling and CNV, thereby limiting the comparability of cohorts as biologically and genetically heterogeneous tumors may have been analyzed [9, 10]. Herein, the use of the integrated molecular-morphological risk classifier would have had significant implications for the clinical management of affected patients. A total of only 15 patients in our cohort received adjuvant radiotherapy, reflecting the ongoing uncertainty regarding the regular use of postoperative treatment of grade 2 meningiomas over the past years [2, 25]. The use of the integrated molecular-morphological risk classifier revealed that four of these patients (26.6%) receiving radiotherapy had a low risk of tumor progression, an insight which could have influenced the patients’ decision making. In total, information of the classifier could have informed the treatment decision making process concerning radiotherapy in over 60% of cases. Moreover, the assumed biological behavior of tumors can also be utilized to adjust the follow-up intervals. High risk tumors warrant close monitoring while low risk grade 2 meningiomas may not necessarily need follow-up every six months as suggested by current guidelines [2]. A risk-adapted management could, therefore, reduce the socioeconomic burden on patients, physicians, and healthcare systems.

These clinical implications highlight the necessity of further investigations, particularly concerning the role of the risk-adapted use of adjuvant radiotherapy. Two prospective interventional studies are aiming to ultimately define the role of adjuvant radiotherapy in grade 2 meningiomas after gross total resection. The two trials, the EORTC 1308/ROAM and NRG BN003, however, have been set up using histopathological criteria, i.e., grading, alone to assess tumor aggressiveness [8]. Our results strongly indicate that molecular-based risk stratification of tumors is highly advisable to study homogeneous patient cohorts. Only by doing so, one can adequately assess the efficacy and safety of an experimental treatment. Therefore, retrospective molecular work-up should be considered to determine the actual biological aggressiveness of the included tumors as suggested by others [26]. Thus, future meningioma trials should consider the use of molecular characteristics to stratify patients in the first place to improve comparability and cohort homogeneity [13].

While the assessment of the methylation profile and incorporation of CNV and histopathological grading have proven valuable in determining the biological aggressiveness and recurrence risk of meningiomas, the broad and consistent implementation of the integrated molecular classifier is resource-intensive [10]. Therefore, alternative methods and prognostic markers that are cost-effective and comparably sensitive are needed. Specific CNV can be a potential solution. Herein, the 1p status demonstrated an excellent correlation with the risk stratification of low and intermediate/high risk tumors per integrated risk grouping (Additional file 1: Fig. S2). This is in accordance with recent analyses, including a retrospective evaluation of patients enrolled in the EORTC 22042–26042 trial [10, 27–29]. This growing evidence should be considered and validated in future research, particularly in prospective clinical trials. While several integrated molecular classifiers for meningiomas exist, there is a distinct need for classifier harmonization and further investigations in distinct subgroups like grad 2 tumors [13].

Moreover, risk factors beyond the methylation class and CNV are well known, such as TERT promoter mutations or homozygous and heterozygous deletions of CDKN2A/B [17, 30–33]. However, the distinct frequencies of such genetic risk factors in the subgroup of grade 2 meningiomas remain of interest. The optimal approaches when to determine these markers are still not clear or debatable [21]. Herein, we screened histologically defined grade 2 meningiomas and only found one TERT promoter mutation and one homozygous CDKN2A/B deletion during our molecular testing (< 1% of analyzed tumors). Notably, both alterations were found in the same high risk tumor (methylation family malignant) which recurred locally only 7.4 months after initial resection. The reported frequency of TERT promoter mutations in histopathologically defined grade 2 tumors was noticeably higher with 5.7% [30]. Likewise, the rate of homozygous CDKN2A/B deletions was approximately 7% in grade 2 meningiomas in another cohort [9, 21]. However, it is important to acknowledge that the observed differences may be attributed to variations in the application of different WHO tumor classifications. In particular, we had to exclude 22 tumors (22/123, 17.8%) during our grading reassessment due to an insufficient number of mitoses, as the specific microscope field size used for assessment must be taken into consideration according to the WHO 2021 classification.

As we have not identified any TERT promoter mutation or homozygous CDKN2A/B deletion in the full intermediate risk group meningiomas (62 cases, data not shown), we share the view of Hielscher and colleagues and do not recommend routine testing for TERT promoter mutations or dedicated assessment of homozygous CDKN2A/B deletions, the latter especially in the absence of methylation analysis [21]. Another topic of interest is the use of different methylation family classifiers, namely the brain tumor classifier v12.5 and the meningioma classifier v2.4. As the methylation family has impact on the final risk group according to the integrated molecular-morphological risk score, its classification is particularly important [10]. We tested both classifiers and found minor differences concerning the subgroups benign and intermediate (Additional file 1: Figs. S11 and 12). However, differences were small and in the final risk group assessment, only six cases differed (6.0%). This finding is in general agreement with previous studies [21]. Therefore, both methylation family classifiers may be used as the chance of a clinically meaningful difference between classifiers is low.

As multiple different genetic and molecular classifiers for meningiomas have been introduced, it will be crucial to combine their characteristics and advantages to ultimately establish one comprehensive classifier which can be prospectively validated and routinely implemented [13]. Meningiomas remain a challenging tumor entity, especially in case of grade 2 and 3 tumors and in the absence of established targeted therapies [2, 3]. The need for treatment individualization remains with various unanswered questions, especially concerning the use of radiotherapy (timing, dose escalation, safety margins, functional imaging for target delineation). This work highlights the fundamental role of the integrated molecular-morphological risk score to unravel and clinically stratify the heterogenous group of grade 2 meningiomas into risk groups. As a consequence of our observed findings, we aim to prospectively integrate risk stratification into daily clinical routine to guide our treatment, patient counseling, and follow-up schedules. Nevertheless, this study has limitations, pertinent to its design. First, the retrospective nature and single-center design of this work may have influenced the included samples and data quality as some clinical data were missing, e.g., the resection status in 8 cases. Moreover, the decision of using adjuvant radiotherapy in this patient cohort was most likely affected by distinct patient preferences or tumor characteristics, e.g., subtotal resection. In addition, the relative lack of patients receiving radiotherapy impairs the adequate assessment of the effect on local control in a homogeneous subgroup. Finally, a longer follow-up is necessary to confirm the long-term implications of the classifier.

## Conclusion

In this analysis, we were able to confirm the importance of stratifying histologically defined grade 2 meningiomas into clinically meaningful groups utilizing an established integrated molecular-morphological risk score. The likelihood of a grade 2 meningioma having an intermediate risk profile was only around 50%. The use of an integrated molecular risk stratification could have influenced and informed more than 60% of treatment decision making processes, especially concerning the use of adjuvant radiotherapy. Utilizing molecular information is crucial to guide the clinical management of patients. The 1p status can provide valuable and cost-effective insights in determining the local recurrence risk of histologically defined grade 2 meningiomas.

### Supplementary Information

**Additional file 1.** Supplementary Files.

## Data Availability

Data will be made available upon reasonable request to the corresponding author.

## Abbreviations

CDKN2A/B: Cyclin-dependent kinase inhibitors 2A/B
CNV: Copy number variation
FFPE: Formalin-fixed paraffin-embedded
MRI: Magnetic resonance imaging
OS: Overall survival
TERT: Telomerase reverse transcriptase
WHO: World Health Organization
