# Correction: Clinical implications of DNA methylation-based integrated classification of histologically defined grade 2 meningiomas

**DOI:** 10.1186/s40478-024-01801-3

**Published:** 2024-06-15

**Authors:** Felix Ehret, Eilís Perez, Daniel Teichmann, Sandra Meier, Carola Geiler, Cosmas Zeus, Helene Franke, Siyer Roohani, David Wasilewski, Julia Onken, Peter Vajkoczy, Leonille Schweizer, David Kaul, David Capper

**Affiliations:** 1 Charité – Universitätsmedizin Berlin, Corporate Member of Freie Universität Berlin and Humboldt-Universität zu Berlin, Department of Radiation Oncology, Berlin, Germany; 2 Charité – Universitätsmedizin Berlin, Germany; German Cancer Consortium (DKTK), partner site Berlin and German Cancer Research Center (DKFZ), Heidelberg, Germany; 3 Charité – Universitätsmedizin Berlin, Corporate Member of Freie Universität Berlin and Humboldt-Universität zu Berlin, Department of Neuropathology, Berlin, Germany; 4 Charité – Universitätsmedizin Berlin, Corporate Member of Freie Universität Berlin and Humboldt-Universität zu Berlin, Berlin School of Integrative Oncology (BSIO), Berlin, Germany; 5 Berlin Institute of Health at Charité – Universitätsmedizin Berlin, BIH Biomedical Innovation Academy, BIH Charité Junior Clinician Scientist Program, Berlin, Germany; 6 Charité – Universitätsmedizin Berlin, Corporate Member of Freie Universität Berlin and Humboldt-Universität zu Berlin, Department of Neurosurgery, Berlin, Germany; 7 Institute of Neurology (Edinger Institute), University Hospital Frankfurt, Goethe University Frankfurt, Frankfurt Am Main, Germany

**Correction: Acta Neuropathologica Communications (2024) 12:74**

10.1186/s40478-024-01739-6

Following publication of the original article [1], the given and family names of Zeus Cosmas were incorrectly structured and the author’s name ‘David Wasilewski’ was incorrectly written as ‘David Wasilewksi’.

The original article has been corrected.
