# Supplementary material for: Clinical implications of DNA methylation-based integrated classification of histologically defined grade 2 meningiomas
Source: Acta Neuropathol Commun. 2024 May 8;12:74. doi: 10.1186/s40478-024-01739-6 (PMC11080225; doi:10.1186/s40478-024-01739-6)

**Supplementary Figure 1.** Case example with the 3 CNV which are relevant for the integrated molecular-morphologic risk score calculation (1p, 6q, 14q losses in red). CNV assessment is done visually and losses will be counted once the loss exceeds 5%.


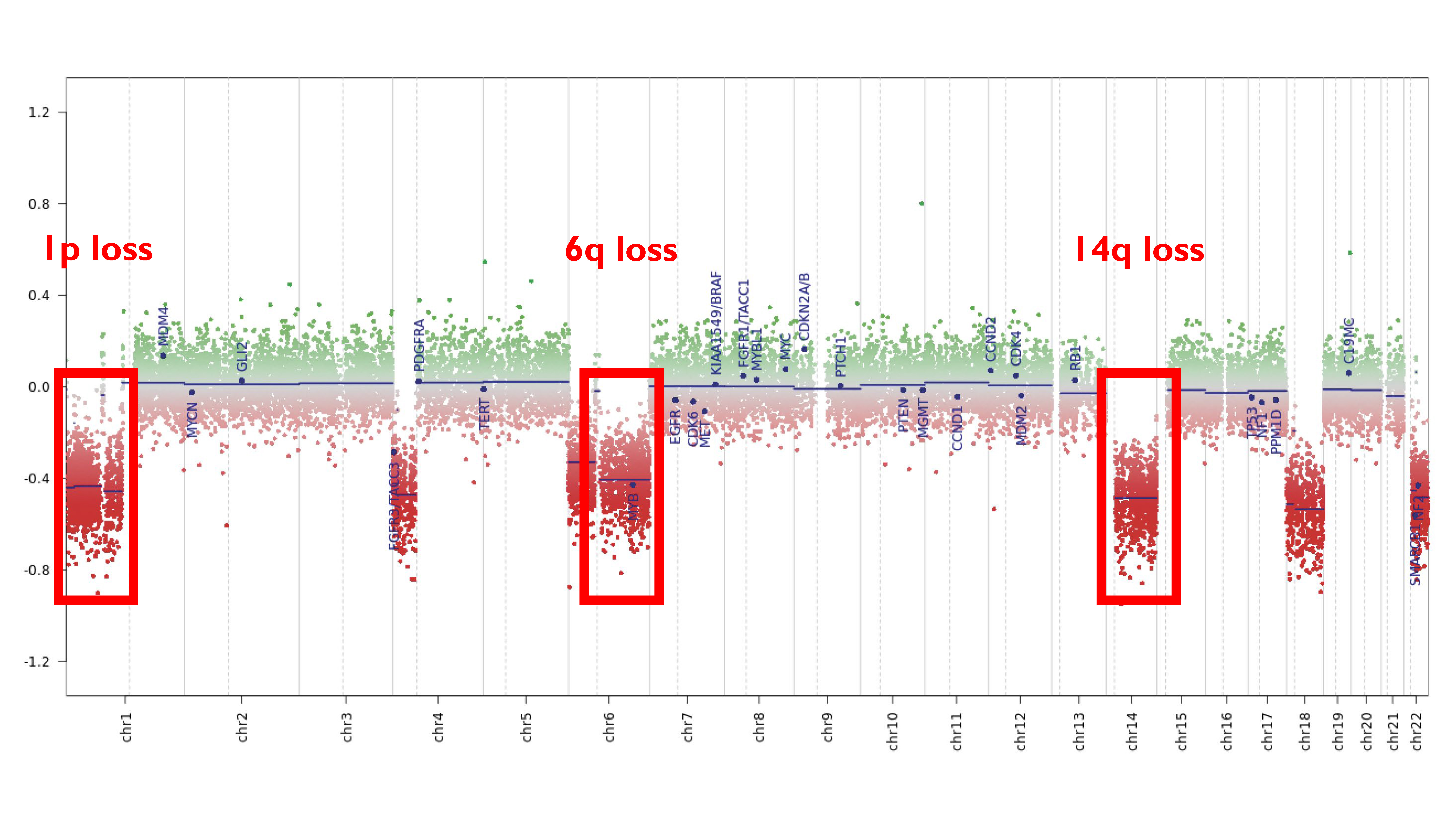


**Supplementary Figure 2.** Comparison of 1p status and integrated risk groups (low vs. intermediate/high) demonstrated nearly identical local control rates among both corresponding subgroups.


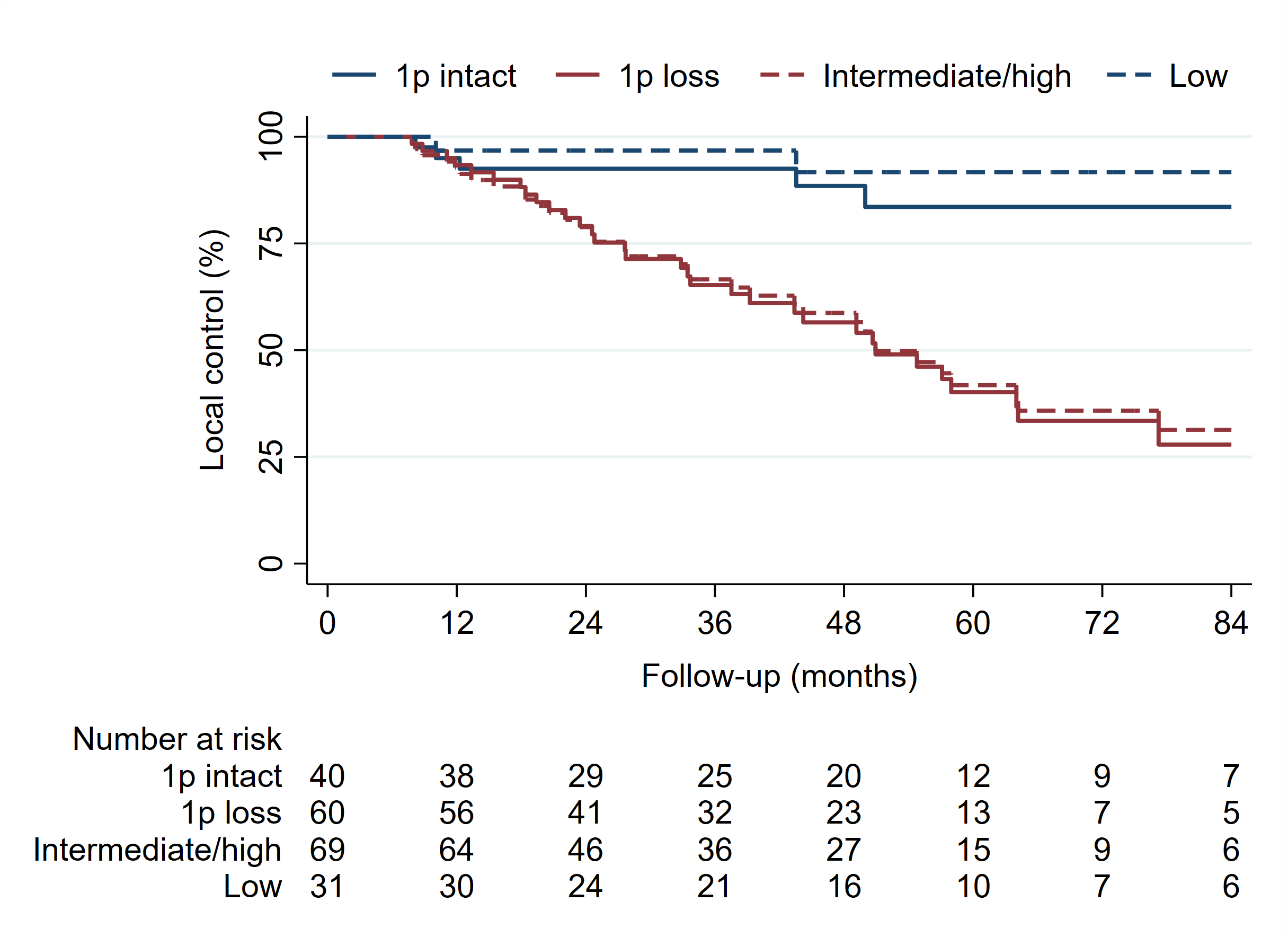


**Supplementary Figure 3.** Local control stratified by resection status (gross total resection vs. subtotal resection). Gross total resections were associated with a decreased risk of local tumor progression in the multivariable Cox regression analysis.


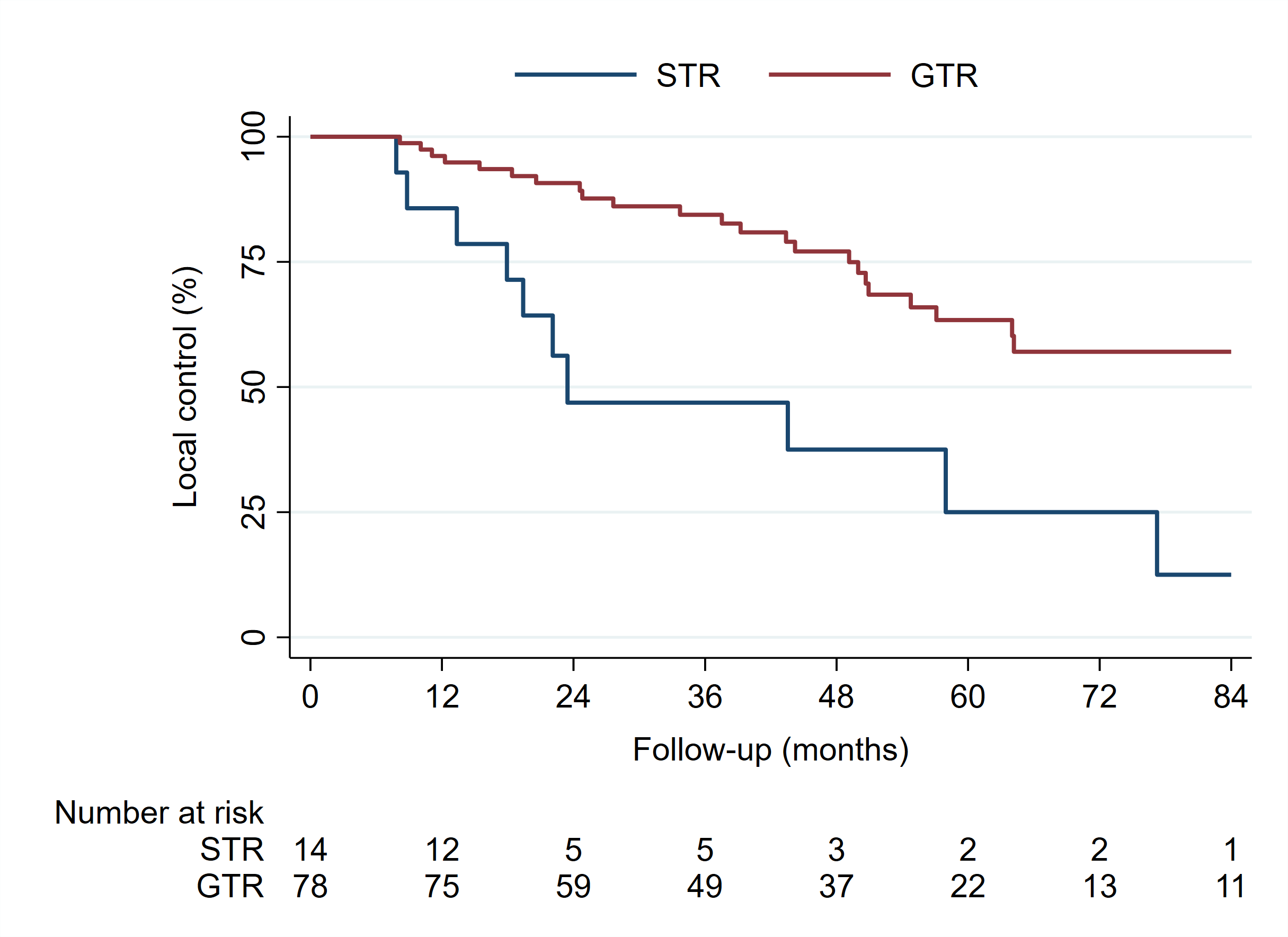


GTR: gross total resection, STR: subtotal resection.

**Supplementary Figure 4.** Local control stratified by adjuvant radiotherapy. While the multivariable Cox regression analysis did not confirm a significant association of postoperative radiotherapy with local control, a visual trend suggesting a favorable long-term control can be observed (Table 2).


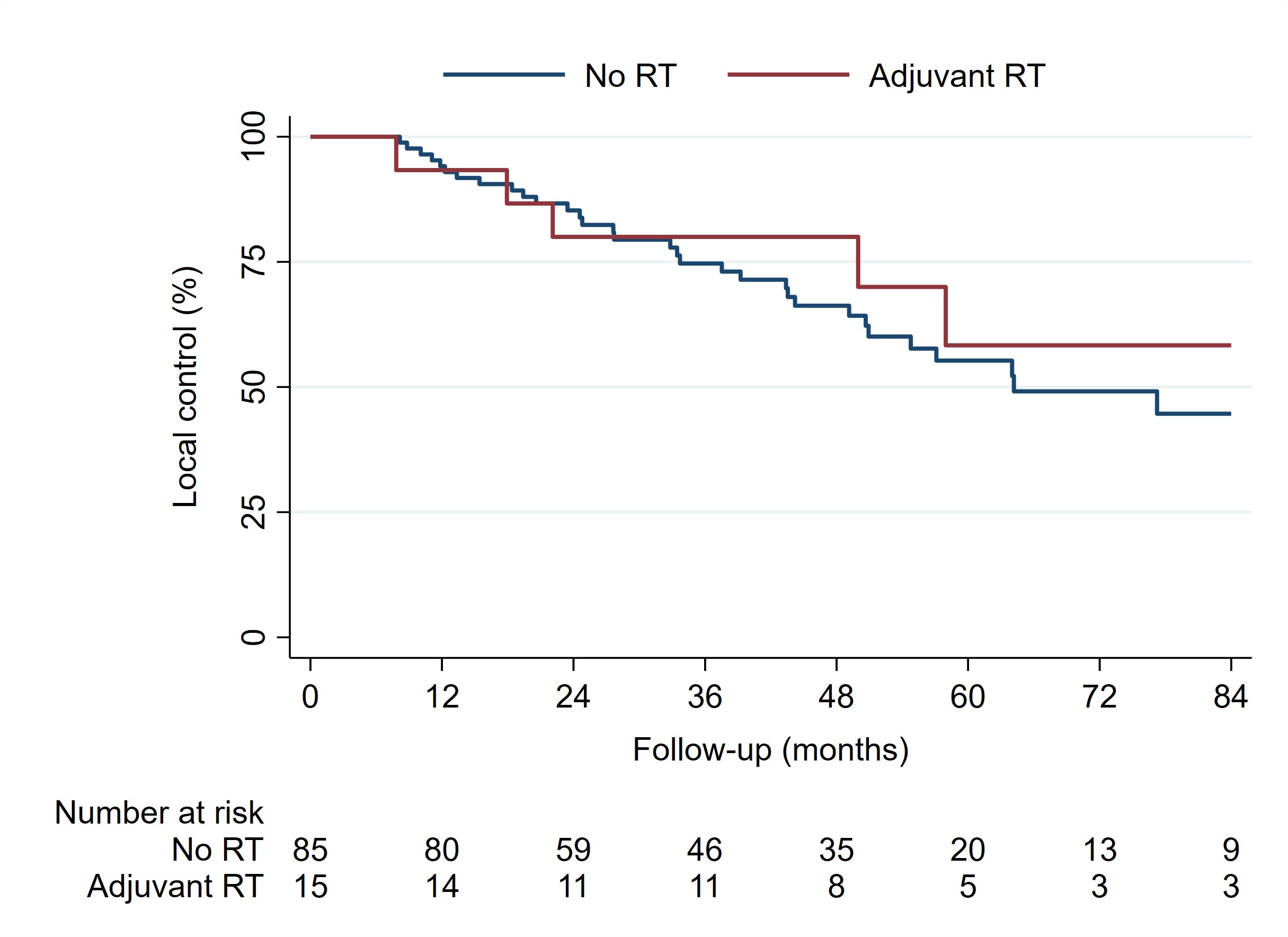


**Supplementary Figure 5.** Progression-free survival of the full cohort.


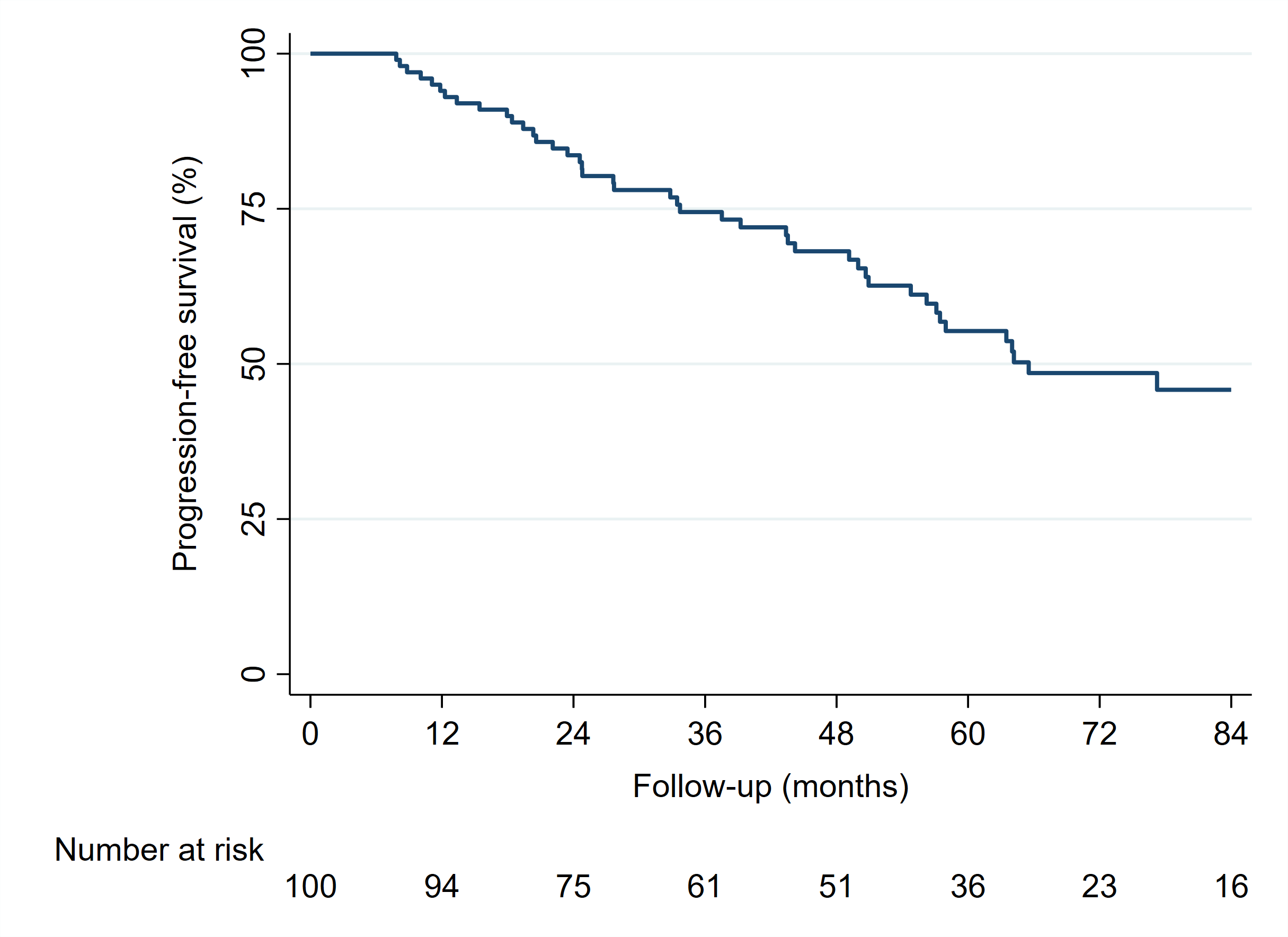


**Supplementary Figure 6.** Progression-free survival stratified by integrated molecular-morphological risk groups. Comparable to the local control rates, intermediate and high risk tumors were found to have a worse progression-free survival rate.


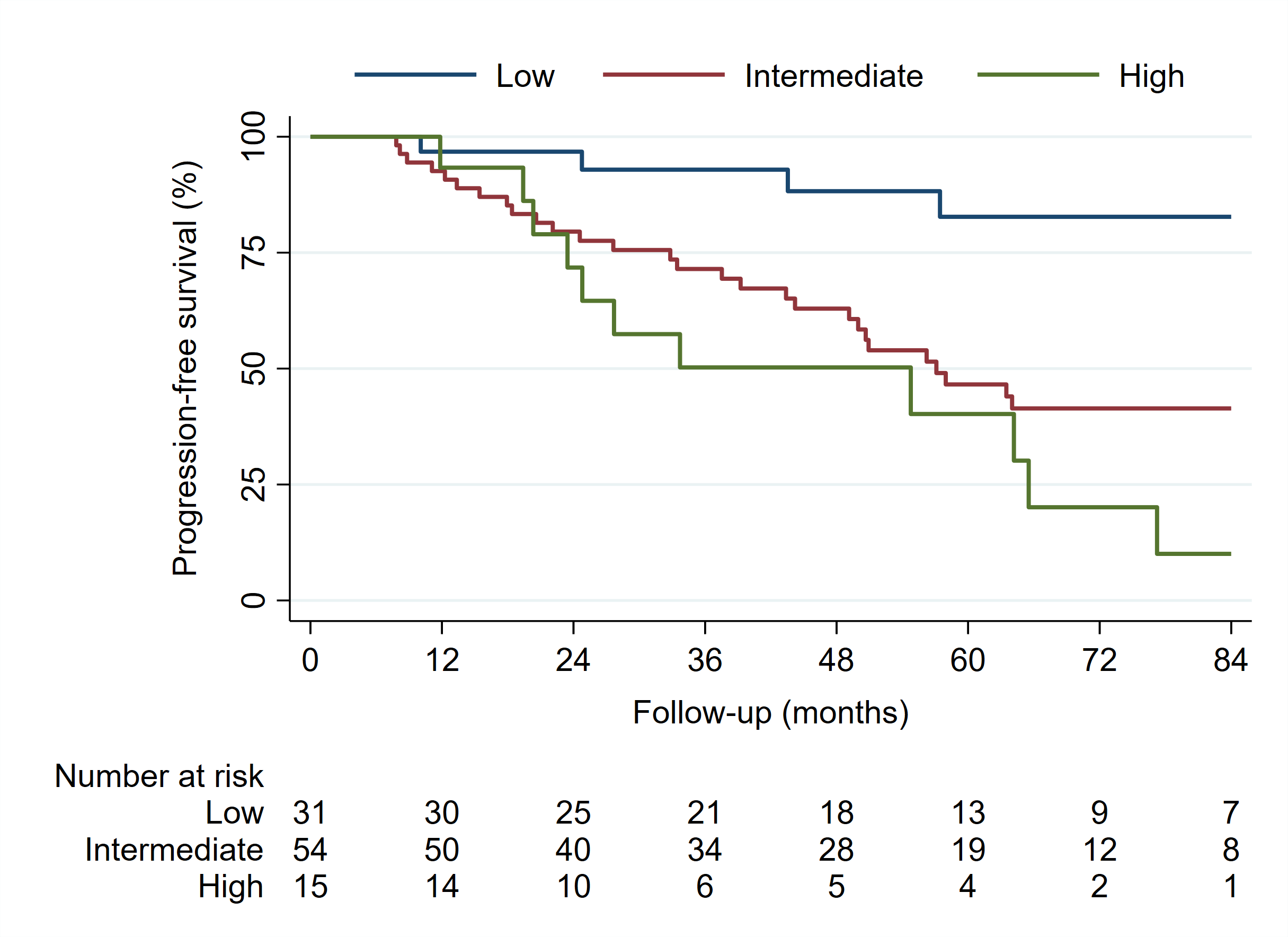


**Supplementary Figure 7.** Overall survival stratified by integrated molecular-morphological risk grouping. Both groups, intermediate and low, showed a favorable and comparable survival rate. The high risk tumors had a markedly worse overall prognosis.


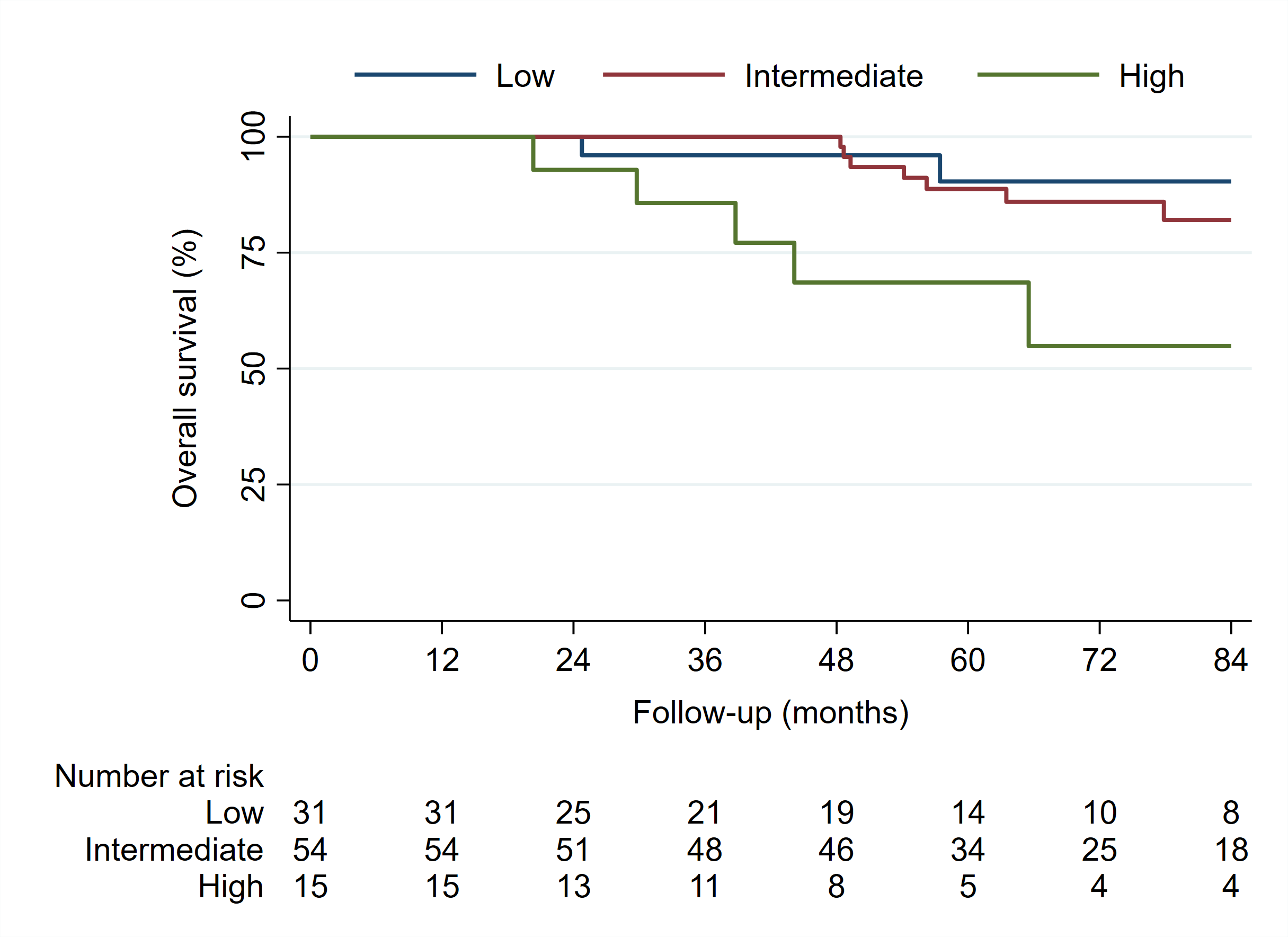


**Supplementary Figure 8.** Local control of low risk tumors stratified by adjuvant radiotherapy. No local tumor progressions were observed in patients undergoing postoperative radiotherapy. However, the local control of cases without adjuvant treatment was still favorable in comparison with intermediate and high risk tumors.


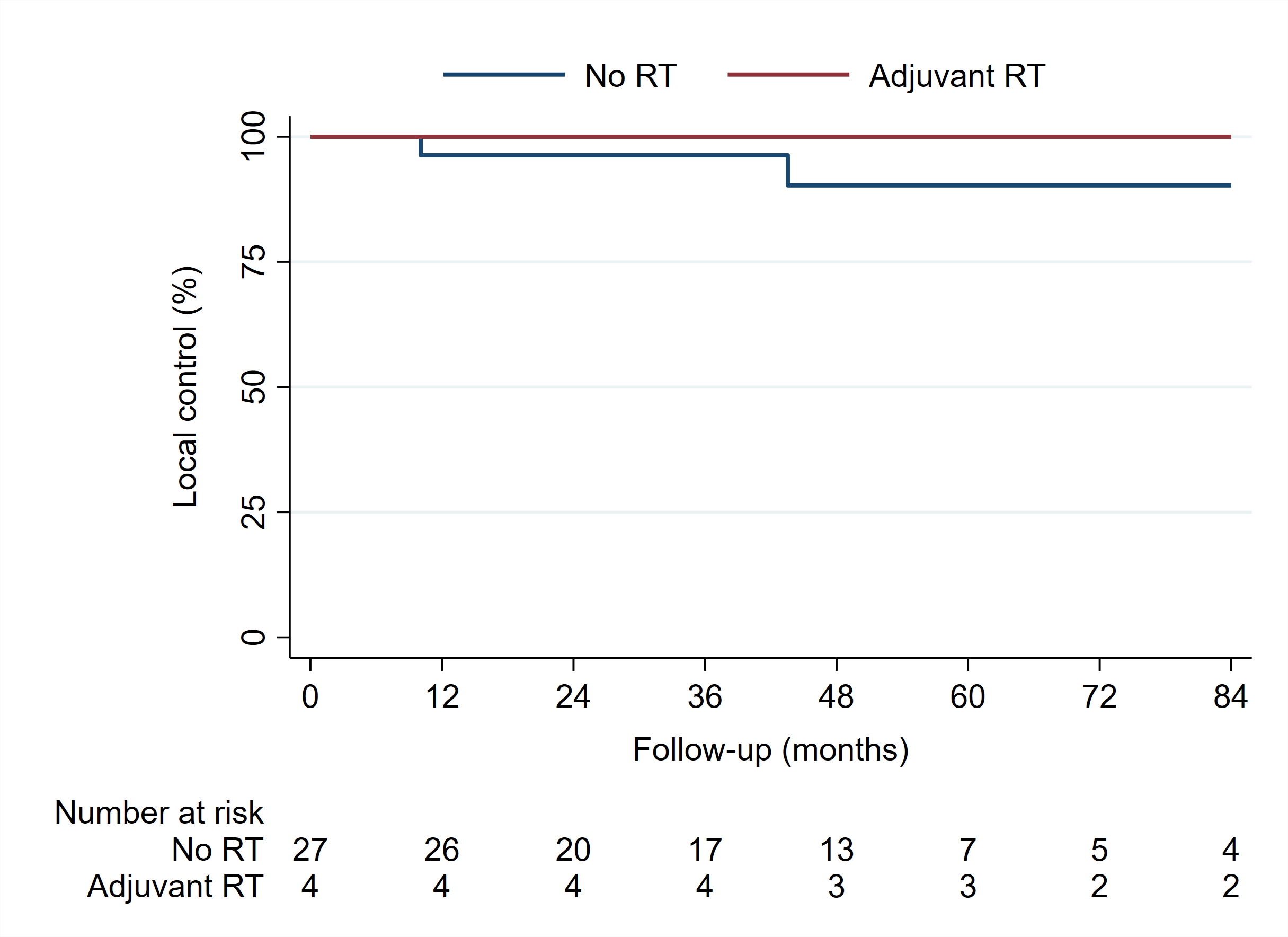


**Supplementary Figure 9.** Local control of intermediate risk tumors stratified by adjuvant radiotherapy. No clear visual benefit can be derived from the use of postoperative radiotherapy. However, the distinct baseline characteristics such as the higher subtotal resection rate in patients with radiotherapy and low overall number of patients with postoperative treatment might mask beneficial radiotherapy effects (5/9 patients (55.5%) with radiotherapy underwent subtotal resection).


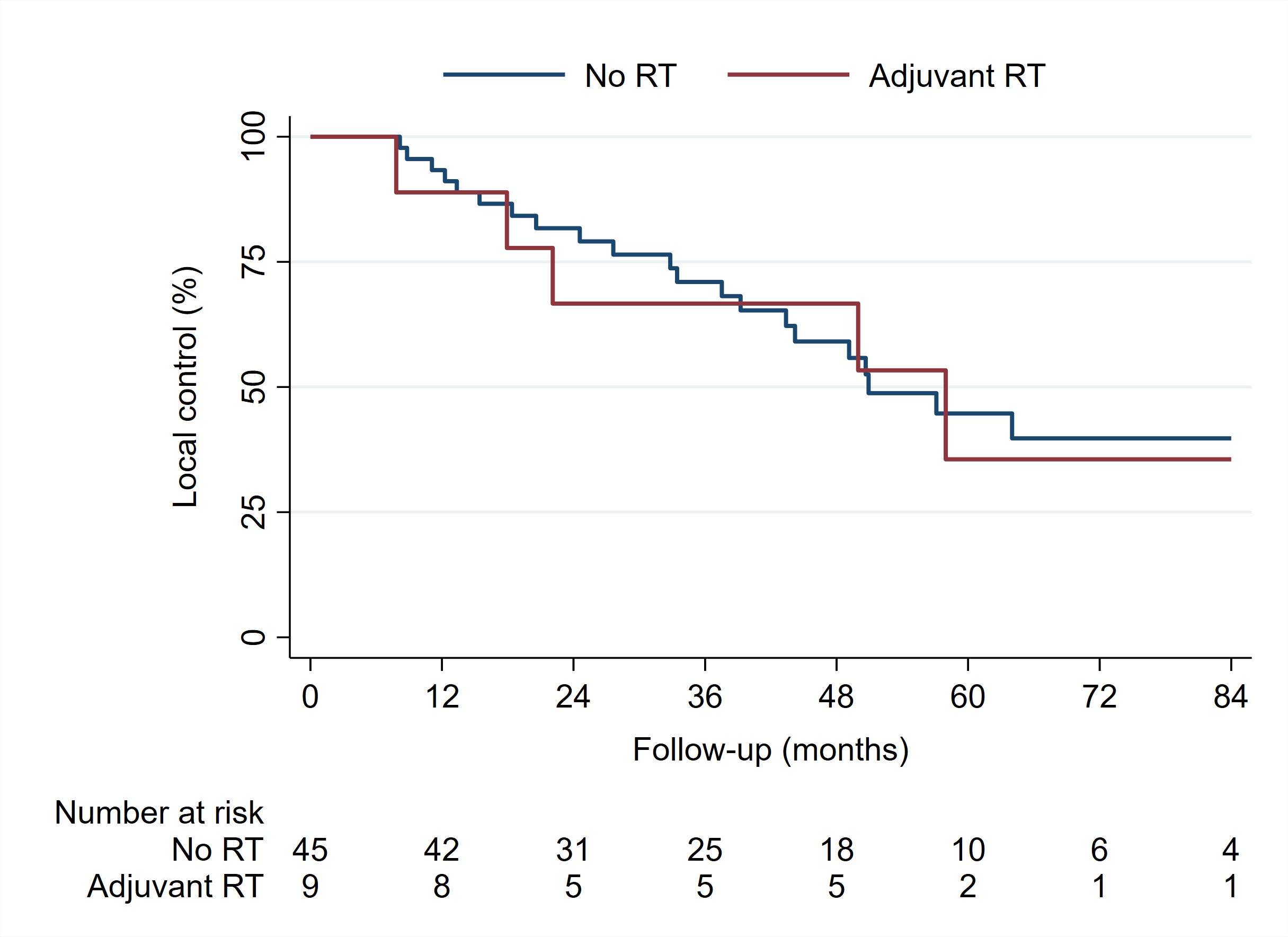


**Supplementary Figure 10.** Local control of high risk tumors stratified by adjuvant radiotherapy. In this subgroup, patients without adjuvant radiotherapy had a poor local control with a median time of less than three years. Both patients with postoperative treatments remained free of local tumor progression.


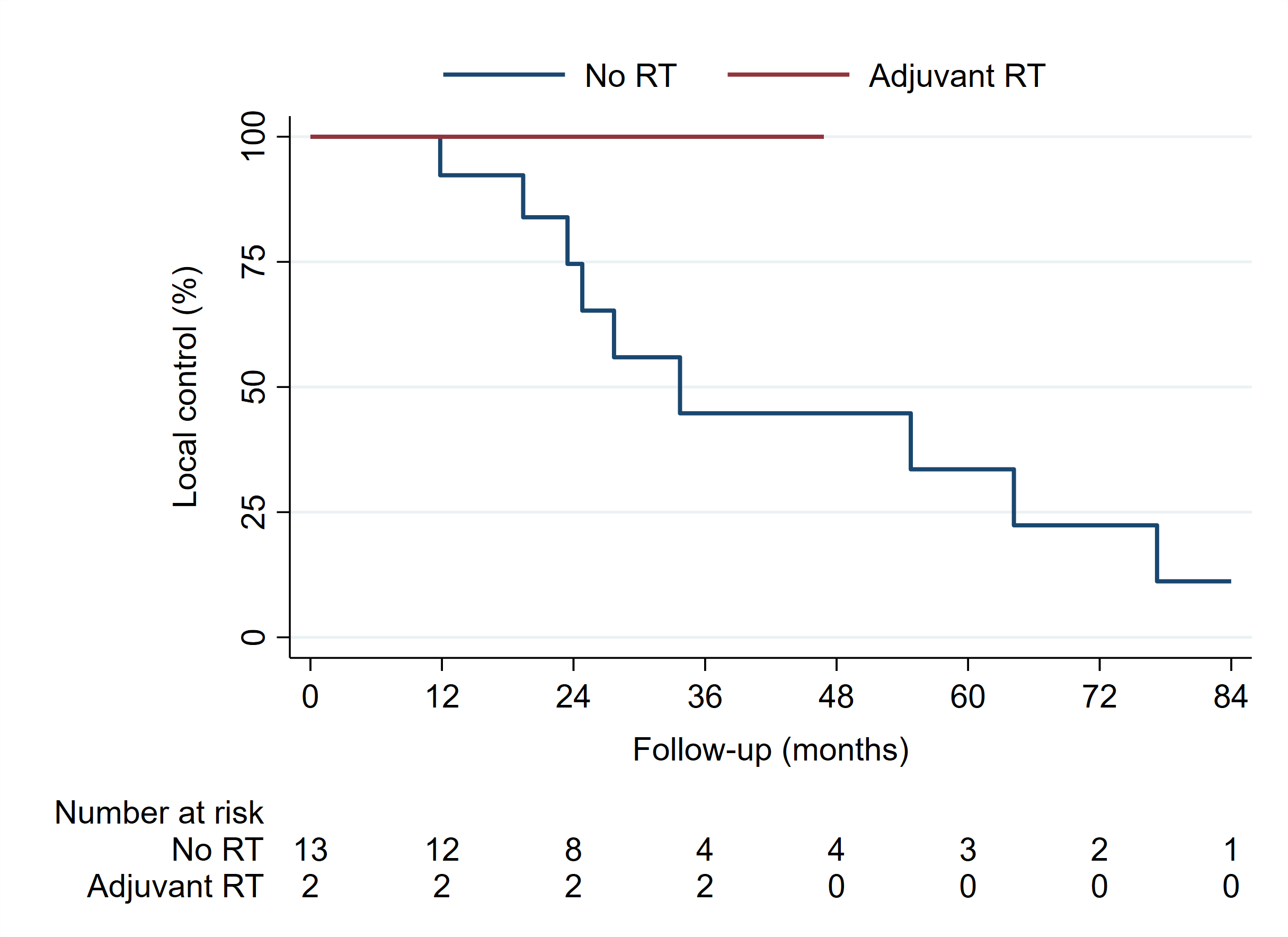


**Supplementary Figure 11.** Local control rates based on the integrated molecular-morphological risk score utilizing the meningioma classifier v2.4. Comparable to the results based on the brain tumor classifier v12.5 (Figure 4), intermediate and high risk tumors show a worse overall local control compared to low risk tumors.


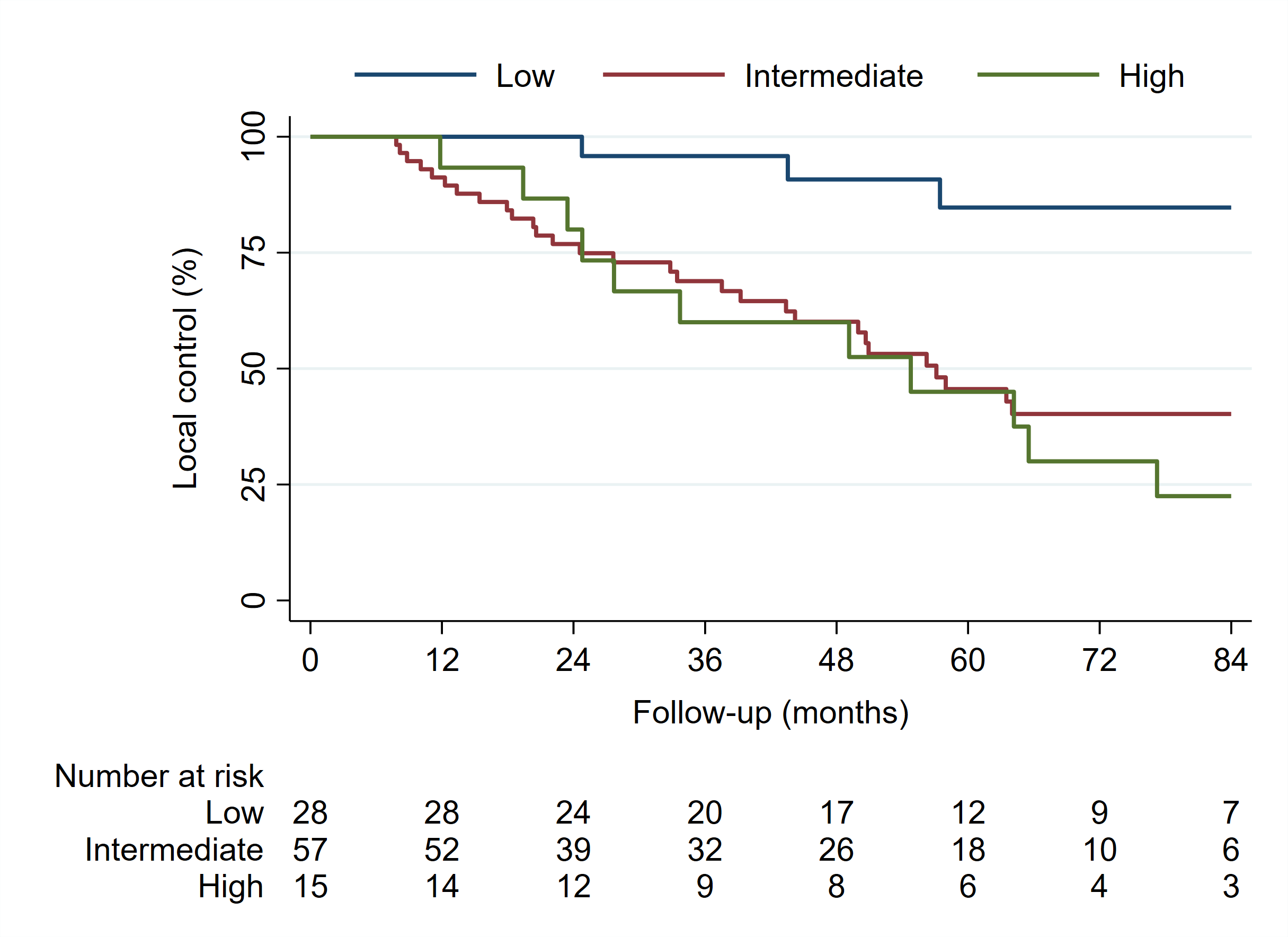


**Supplementary Figure 12.** Alluvial plot for the assessment of the integrated molecular-morphological risk group using the methylation families based on the meningioma classifier v2.4.


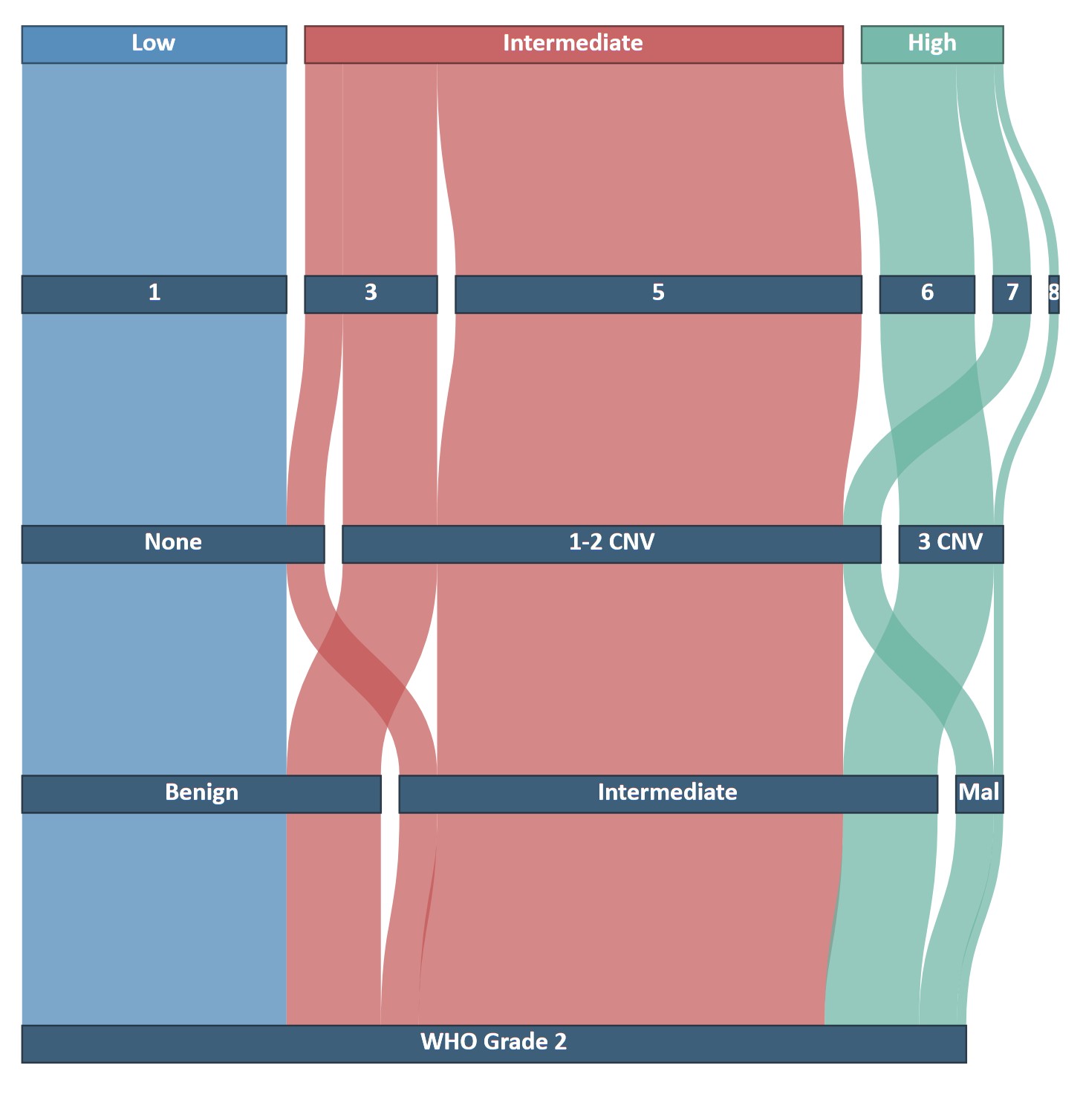

Supplement: Supplementary file 1 — Additional file 1. Supplementary Files. [file 40478_2024_1739_MOESM1_ESM.docx]
